# 2-(4-Chloro­phen­yl)-1,3-dioxane – localization of hydrogen atoms

**DOI:** 10.1107/S2414314625010466

**Published:** 2025-11-28

**Authors:** Eric Cyriel Hosten, Richard Betz

**Affiliations:** a Nelson Mandela University, Summerstrand Campus, Department of Chemistry, University Way, Summerstrand, PO Box 77000, Port Elizabeth, 6031, South Africa; Goethe-Universität Frankfurt, Germany

**Keywords:** crystal structure, C—H⋯O contacts

## Abstract

The title compound, C_10_H_11_ClO_2_, is an acetal-protected derivative of 4-chloro­benzaldehyde. The aliphatic ring adopts a chair conformation. In the crystal, weak C—H⋯O contacts connect the mol­ecules into centrosymmetric dimers.

## Abstract 2

The title compound is the acetal of 4-chloro­benzaldehyde and 1,3-propane­diol. In the crystal, weak C—H⋯O contacts connect the mol­ecules to centrosymmetric dimers.

## Graphical abstract

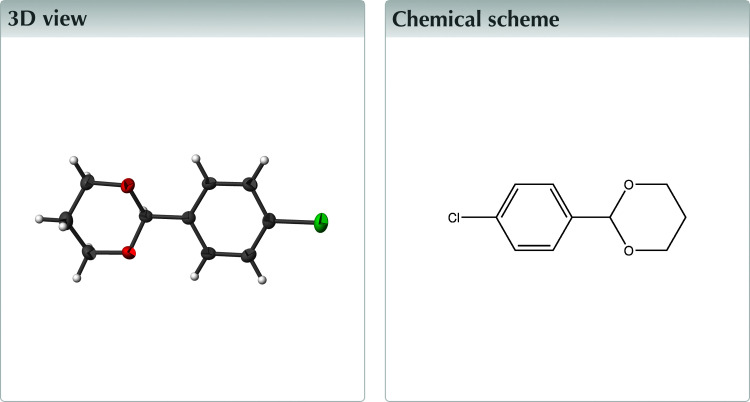

## Structure description

Aldehydes are one of the most important synthons in preparative organic chemistry on grounds of their versatile redox and nucleophilic properties that can be exploited for the synthesis of alcohols, carb­oxy­lic acids as well as a wide variety of additives such as, among others, sulfites and cyano- or chloro­hydrines that, in their own right, are crucial building blocks for the synthesis of other target compounds (Becker *et al.*, 2000 ▸). Owing to the reactivity of the CHO as well as the keto functional groups, protecting them during crucial reaction sequences in the wake of multi-step synthesis is of paramount importance. One common way to achieve this is by converting the carbonyl functionality to an acetal moiety by condensation with alcohols. Of particular inter­est are ring-type acetals derived from diols as these can give rise to inter­esting conformations of the resulting heterocycle. For cyclic acetals derived from benzladehyde, it might be inter­esting to elucidate whether the nature of substituents on the aromatic core might be able to determine the conformation of the dioxolane moiety. In this context, structural information about cyclic acetals with five-membered dioxolane rings derived from benzaldehyde derivatives bearing one halogen substituent in an *ortho* position (DeAngelis *et al.*, 2008 ▸; Li *et al.*, 2008 ▸; Liu *et al.*, 2009 ▸) or *para* position (Bentabed-Ababsa *et al.*, 2008 ▸; Gildenast *et al.*, 2023 ▸; Wang *et al.*, 2009 ▸; Bentabed-Ababsa *et al.*, 2009 ▸; Toda *et al.*, 2022 ▸; Yuan *et al.*, 2017*a* ▸; Bhaumik *et al.*, 2017 ▸) is available next to metrical parameters derived by diffraction studies based on single crystals for six-membered dioxolane rings derived from benzaldehyde derivatives bearing one halogen substituent in an *ortho* position [Imamoto *et al.*, 1984 ▸; Laing *et al.*, 1984 ▸; Wang *et al.*, 2010 ▸; Sun *et al.*, 2010 ▸; Guang-Chuan *et al.*, 2019 ▸; Qiong *et al.*, 2019 ▸; Mezo *et al.*, 2021 ▸; Yuan *et al.*, 2016*a* ▸,*b* ▸, 2018 ▸; Brown *et al.*, 1990 ▸; Ng *et al.*, 2006 ▸; Li *et al.*, 2016 ▸; Jia *et al.*, 2012 ▸, 2016*a* ▸; Ou *et al.*, 2018 ▸; Warwicker, 1961 ▸ (no three-dimensional coordinates deposited)], *meta* position (Li *et al.*, 2011 ▸; Ishihara *et al.*, 2021 ▸) or *para* position (Hertung *et al.*, 1981 ▸; Eliel *et al.*, 1976 ▸; de Kok & Romers, 1970 ▸; Thuaud *et al.*, 2016 ▸; Zhang *et al.*, 2013 ▸, 2014 ▸, 2016 ▸; Benhamou *et al.*, 2019 ▸; Scheffler & Mahrwald, 2012 ▸; Yuan *et al.*, 2017*b* ▸; Zou *et al.*, 2021 ▸; Jia *et al.*, 2016*b* ▸; Janner *et al.*, 2022 ▸; Xu *et al.*, 2019 ▸). In continuation of our inter­est in the structural chemistry of acetals (Betz & Klüfers, 2007*a* ▸,*b* ▸,*c* ▸; Betz & Klüfers, 2008 ▸; Betz *et al.*, 2007*a* ▸,*b* ▸,*c* ▸,*d* ▸), we synthesized the title compound and determined its mol­ecular and crystal structure. Although the latter has been reported previously (de Kok & Romers, 1970 ▸; CSD ref code: CPDIOX), no hydrogen atoms were taken into account during the refinement, thus precluding the possibility to analyse the intra- and inter­molecular contacts. This study is intended to close this gap.

**Figure 1:**
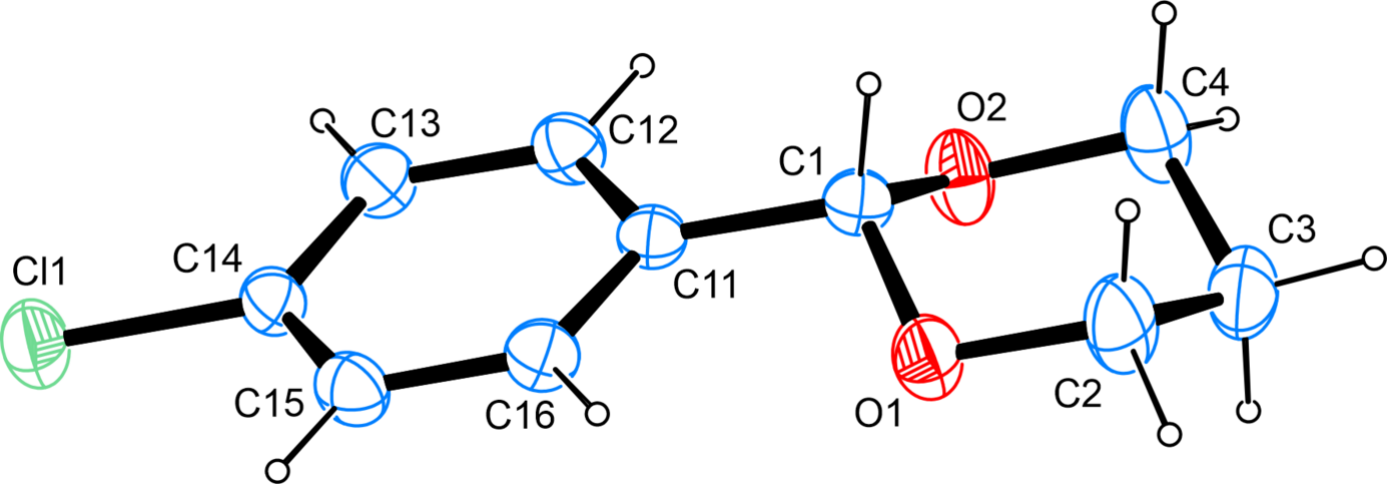
The mol­ecular structure of the title compound, with atom labels and anisotropic displacement ellipsoids (drawn at 50% probability level).

The structure solution shows the presence of the 1,3-propane­diol derived cyclic acetal of 4-chloro­benzaldehyde. C—O bond lengths and angles are found in good agreement with other cyclic acetals whose metrical parameters have been deposited with the Cambridge Structural Database (Groom *et al.*, 2016 ▸). A conformational analysis (Cremer & Pople, 1975 ▸) of the 1,3-dioxolane ring shows the latter to adopt a ^4^*C*_1_ (^C4^*C*_O1_) conformation (Boeyens, 1978 ▸). The least-squares planes as defined by the non-hydrogen atoms of the aromatic moiety on the one hand and the oxolane moiety on the other hand inter­sect at an angle of 18.35 (7)° only (Fig. 1 ▸).

**Figure 2:**
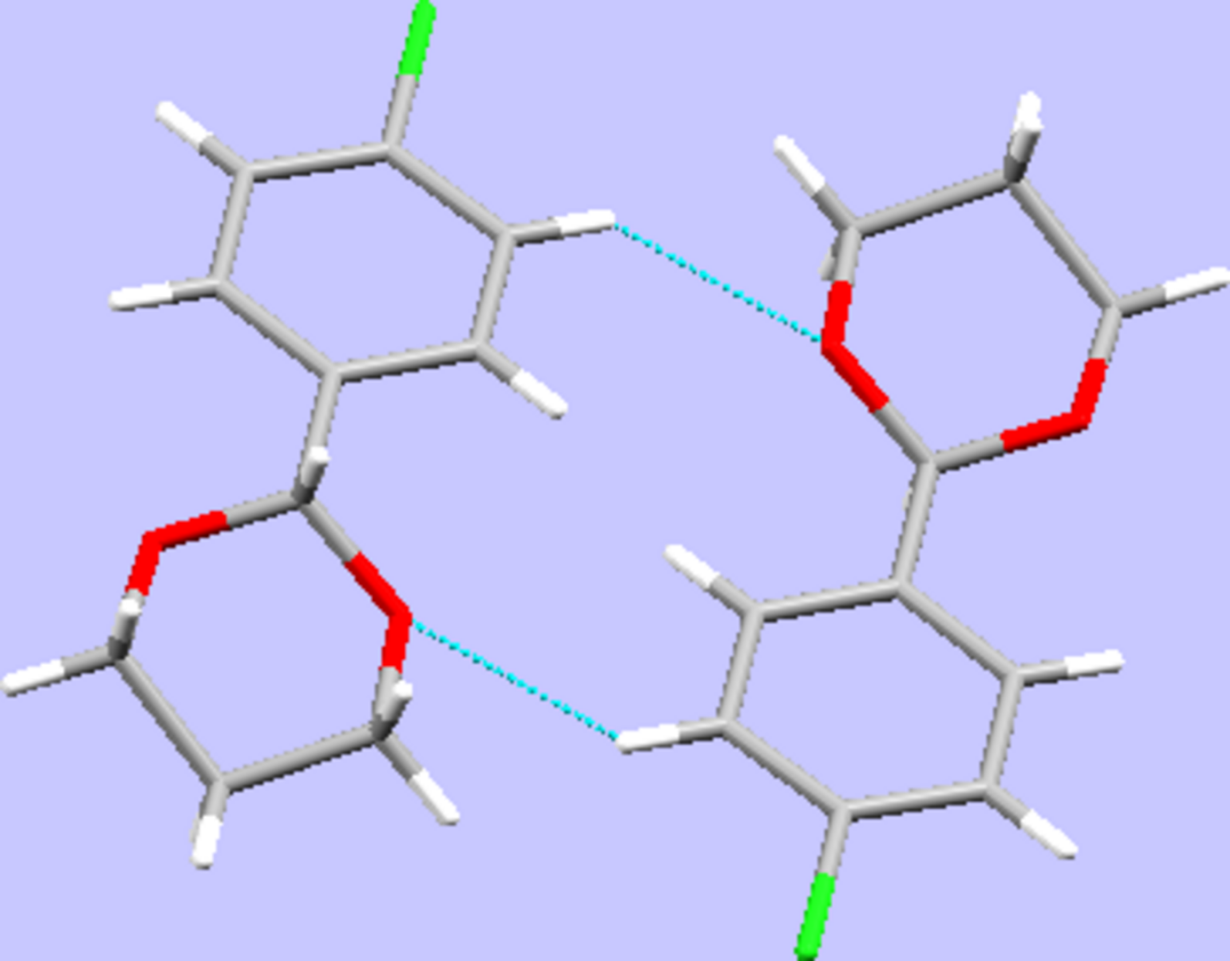
Inter­molecular contacts, viewed along [100].

**Figure 3:**
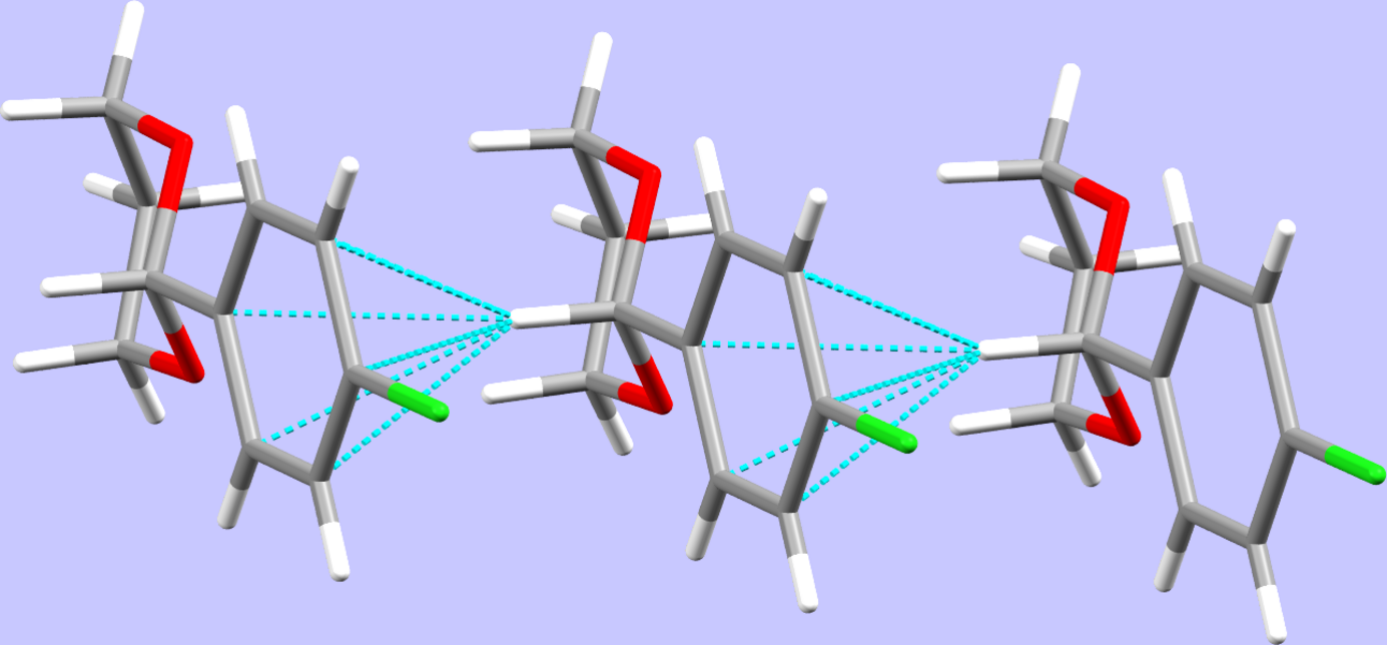
Inter­molecular C—H⋯π contacts, viewed along [010].

In the crystal, only weak C—H⋯O contacts are observed whose range falls by 0.06 Å below the sum of van-der Waals radii of the atoms participating in them. These are established between one of the hydrogen atoms in an *ortho* position to the chlorine atom on the aromatic moiety as donor and one of the intra­cyclic oxygen atoms as acceptor, giving rise to centrosymmetric dimers (Fig. 2 ▸, Table 1 ▸). In terms of graph-set analysis, (Etter *et al.*, 1990 ▸; Bernstein *et al.*, 1995 ▸) these contacts require a 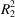
(12) descriptor on the unary level. While π-stacking is not a prominent stabilizing feature with the shortest inter­centroid distance between two centres of gravity measured at 4.7025 (8) Å (the length of the crystallographic *a* axis), a C—H⋯π contact is present that is supported by the hydrogen atom of the methine group. Furthermore, a C—Cl⋯π contact is apparent. Although the latter exhibits an angle of 90.78 (5)° (far away from linearity), it has been found that this does not significantly weaken the force of such an inter­action (Imai *et al.*, 2008 ▸). Both inter­actions involving the aromatic system result in connecting the mol­ecules to chains along the crystallographic *a-*axis direction (Fig. 3 ▸, Table 1 ▸).

## Synthesis and crystallization

The compound was prepared following a standard procedure by reacting *para*-chloro­benzaldehyde with 1,3-propane­diol (Dong *et al.*, 2018 ▸). Crystals suitable for the diffraction study were obtained upon storing the isolated product at ambient conditions.

## Refinement

Crystal data, data collection and structure refinement details are summarized in Table 2 ▸.

## Supplementary Material

Crystal structure: contains datablock(s) I. DOI: 10.1107/S2414314625010466/bt4190sup1.cif

Structure factors: contains datablock(s) I. DOI: 10.1107/S2414314625010466/bt4190Isup2.hkl

Supporting information file. DOI: 10.1107/S2414314625010466/bt4190Isup3.cml

CCDC reference: 2504999

Additional supporting information:  crystallographic information; 3D view; checkCIF report

## Figures and Tables

**Table 1:** Hydrogen-bond geometry (Å, °) *C*_g_(1) is the centroid of carbon atoms C11–C16.

*D*—H⋯*A*	*D*—H	H⋯*A*	*D*⋯*A*	*D*—H⋯*A*
C13—H13⋯O2^i^	0.95	2.66	3.4686 (16)	143
C1—H1⋯*C*_g_(1)^ii^	1.00	2.58	3.5282 (15)	158
C14—Cl1⋯*C*_g_(1)^iii^	1.74 (1)	3.49 (1)	3.9197 (15)	91 (1)

Symmetry codes: (i) 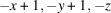; (ii) 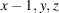; (iii) 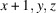.

**Table 2:** Experimental details

Crystal data	
Chemical formula	C_10_H_11_ClO_2_
*M*_r_	198.64
Crystal system, space group	Triclinic, *P* 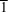
Temperature (K)	200
*a*, *b*, *c* (Å)	4.7025 (1), 9.6336 (3), 10.8933 (3)
α, β, γ (°)	102.9908 (9), 90.6153 (10), 103.538 (1)
*V* (Å^3^)	466.45 (2)
*Z*	2
Radiation type	Mo *K*α
μ (mm^−1^)	0.37
Crystal size (mm)	0.38 × 0.14 × 0.05

Data collection	
Diffractometer	Bruker APEXII CCD
Absorption correction	Numerical (*SADABS*; Krause *et al.*, 2015 ▸)
*T*_min_, *T*_max_	0.919, 0.996
No. of measured, independent and observed [*I* > 2σ(*I*)] reflections	31279, 2315, 2069
*R*_int_	0.027
(sin θ/λ)_max_ (Å^−1^)	0.667

Refinement	
*R*[*F*^2^ > 2σ(*F*^2^)], *wR*(*F*^2^), *S*	0.032, 0.084, 1.08
No. of reflections	2315
No. of parameters	119
H-atom treatment	H-atom parameters constrained
Δρ_max_, Δρ_min_ (e Å^−3^)	0.40, −0.21

Computer programs: *APEX2* and *SAINT* (Bruker, 2014 ▸), *SHELXS97* (Sheldrick 2008 ▸), *ORTEP-3 for Windows* (Farrugia, 2012 ▸), *Mercury* (Macrae *et al.*, 2008 ▸), *SHELXL2019/3* (Sheldrick, 2015 ▸) and *PLATON* (Spek, 2020 ▸).

## full crystallographic data

### 2-(4-Chlorophenyl)-1,3-dioxane Crystal data

**Table:**

C_10_H_11_ClO_2_	*Z* = 2
*M**_r_* = 198.64	*F*(000) = 208
Triclinic, *P*1	*D*_x_ = 1.414 Mg m^−^^3^
*a* = 4.7025 (1) Å	Mo *K*α radiation, λ = 0.71073 Å
*b* = 9.6336 (3) Å	Cell parameters from 9212 reflections
*c* = 10.8933 (3) Å	θ = 2.2–28.3°
α = 102.9908 (9)°	µ = 0.37 mm^−^^1^
β = 90.6153 (10)°	*T* = 200 K
γ = 103.538 (1)°	Rod, colourless
*V* = 466.45 (2) Å^3^	0.38 × 0.14 × 0.05 mm

### 2-(4-Chlorophenyl)-1,3-dioxane Data collection

**Table:**

Bruker APEXII CCD diffractometer	2069 reflections with *I* > 2σ(*I*)
φ and ω scans	*R*_int_ = 0.027
Absorption correction: numerical (SADABS; Krause *et al.*, 2015)	θ_max_ = 28.3°, θ_min_ = 2.2°
*T*_min_ = 0.919, *T*_max_ = 0.996	*h* = −6→6
31279 measured reflections	*k* = −12→12
2315 independent reflections	*l* = −14→14

### 2-(4-Chlorophenyl)-1,3-dioxane Refinement

**Table:**

Refinement on *F*^2^	Secondary atom site location: difference Fourier map
Least-squares matrix: full	Hydrogen site location: inferred from neighbouring sites
*R*[*F*^2^ > 2σ(*F*^2^)] = 0.032	H-atom parameters constrained
*wR*(*F*^2^) = 0.084	*w* = 1/[σ^2^(*F*_o_^2^) + (0.0328*P*)^2^ + 0.2109*P*] where *P* = (*F*_o_^2^ + 2*F*_c_^2^)/3
*S* = 1.08	(Δ/σ)_max_ = 0.001
2315 reflections	Δρ_max_ = 0.40 e Å^−^^3^
119 parameters	Δρ_min_ = −0.21 e Å^−^^3^
0 restraints	Extinction correction: *SHELXL2019/2* (Sheldrick, 2015), Fc^*^=kFc[1+0.001xFc^2^λ^3^/sin(2θ)]^-1/4^
Primary atom site location: structure-invariant direct methods	Extinction coefficient: 0.009 (3)

### 2-(4-Chlorophenyl)-1,3-dioxane Special details

**Table:**

Geometry. All esds (except the esd in the dihedral angle between two l.s. planes) are estimated using the full covariance matrix. The cell esds are taken into account individually in the estimation of esds in distances, angles and torsion angles; correlations between esds in cell parameters are only used when they are defined by crystal symmetry. An approximate (isotropic) treatment of cell esds is used for estimating esds involving l.s. planes.
Refinement. The carbon-bound H atoms were placed in calculated positions (C–H 0.95 Å for aromatic carbon atoms, C–H 0.99 Å for the methylene groups, C–H 1.00 Å for the methine group) and were included in the refinement in the riding model approximation, with *U*(H) set to 1.2*U*_eq_(C).

### 2-(4-Chlorophenyl)-1,3-dioxane Fractional atomic coordinates and isotropic or equivalent isotropic displacement parameters (Å^2^)

**Table:**

	*x*	*y*	*z*	*U*_iso_*/*U*_eq_	
Cl1	0.71337 (8)	0.09478 (4)	0.16147 (3)	0.03644 (12)	
O1	0.1662 (2)	0.66451 (11)	0.39209 (8)	0.0344 (2)	
O2	0.1908 (2)	0.67841 (10)	0.18198 (9)	0.0312 (2)	
C1	0.0987 (3)	0.58707 (13)	0.26568 (11)	0.0228 (2)	
H1	−0.117911	0.545491	0.251496	0.027*	
C11	0.2512 (3)	0.46337 (13)	0.23963 (11)	0.0222 (2)	
C12	0.3153 (3)	0.40527 (14)	0.11735 (11)	0.0261 (3)	
H12	0.261482	0.443755	0.049967	0.031*	
C13	0.4571 (3)	0.29161 (14)	0.09245 (12)	0.0278 (3)	
H13	0.501563	0.252535	0.008795	0.033*	
C14	0.5326 (3)	0.23623 (13)	0.19160 (12)	0.0251 (2)	
C15	0.4683 (3)	0.29100 (14)	0.31383 (12)	0.0285 (3)	
H15	0.520747	0.251661	0.380890	0.034*	
C16	0.3257 (3)	0.40451 (14)	0.33715 (11)	0.0264 (3)	
H16	0.278748	0.442234	0.420707	0.032*	
C2	0.0114 (4)	0.77863 (17)	0.42187 (14)	0.0410 (4)	
H2A	0.061919	0.833436	0.510809	0.049*	
H2B	−0.202458	0.734805	0.411623	0.049*	
C3	0.0936 (4)	0.88251 (16)	0.33514 (15)	0.0397 (3)	
H3A	−0.025577	0.956148	0.350172	0.048*	
H3B	0.303009	0.934975	0.351864	0.048*	
C4	0.0379 (4)	0.79369 (16)	0.20015 (13)	0.0362 (3)	
H4A	−0.175037	0.750684	0.180963	0.043*	
H4B	0.106251	0.858277	0.141982	0.043*	

### 2-(4-Chlorophenyl)-1,3-dioxane Atomic displacement parameters (Å^2^)

**Table:**

	*U*^11^	*U*^22^	*U*^33^	*U*^12^	*U*^13^	*U*^23^
Cl1	0.0357 (2)	0.03054 (18)	0.0467 (2)	0.01563 (14)	0.00267 (14)	0.00859 (14)
O1	0.0519 (6)	0.0352 (5)	0.0206 (4)	0.0236 (5)	−0.0002 (4)	0.0023 (4)
O2	0.0418 (5)	0.0319 (5)	0.0283 (5)	0.0185 (4)	0.0123 (4)	0.0139 (4)
C1	0.0233 (6)	0.0251 (6)	0.0199 (5)	0.0056 (4)	0.0021 (4)	0.0055 (4)
C11	0.0201 (5)	0.0236 (5)	0.0223 (6)	0.0040 (4)	0.0012 (4)	0.0057 (4)
C12	0.0292 (6)	0.0303 (6)	0.0210 (6)	0.0097 (5)	0.0015 (5)	0.0077 (5)
C13	0.0299 (6)	0.0307 (6)	0.0233 (6)	0.0102 (5)	0.0028 (5)	0.0045 (5)
C14	0.0209 (6)	0.0230 (5)	0.0314 (6)	0.0058 (4)	0.0004 (5)	0.0059 (5)
C15	0.0310 (7)	0.0301 (6)	0.0271 (6)	0.0084 (5)	0.0000 (5)	0.0114 (5)
C16	0.0299 (6)	0.0288 (6)	0.0210 (6)	0.0071 (5)	0.0024 (5)	0.0070 (5)
C2	0.0614 (10)	0.0412 (8)	0.0269 (7)	0.0300 (7)	0.0065 (6)	0.0030 (6)
C3	0.0503 (9)	0.0292 (7)	0.0413 (8)	0.0169 (6)	0.0014 (7)	0.0043 (6)
C4	0.0496 (9)	0.0356 (7)	0.0329 (7)	0.0233 (6)	0.0081 (6)	0.0137 (6)

### 2-(4-Chlorophenyl)-1,3-dioxane Geometric parameters (Å, º)

**Table:**

Cl1—C14	1.7419 (12)	C14—C15	1.3821 (18)
O1—C1	1.4041 (14)	C15—C16	1.3898 (18)
O1—C2	1.4363 (16)	C15—H15	0.9500
O2—C1	1.4085 (14)	C16—H16	0.9500
O2—C4	1.4380 (15)	C2—C3	1.515 (2)
C1—C11	1.5056 (16)	C2—H2A	0.9900
C1—H1	1.0000	C2—H2B	0.9900
C11—C16	1.3888 (16)	C3—C4	1.511 (2)
C11—C12	1.3896 (17)	C3—H3A	0.9900
C12—C13	1.3891 (17)	C3—H3B	0.9900
C12—H12	0.9500	C4—H4A	0.9900
C13—C14	1.3844 (18)	C4—H4B	0.9900
C13—H13	0.9500		
			
C1—O1—C2	110.20 (10)	C16—C15—H15	120.5
C1—O2—C4	110.26 (10)	C11—C16—C15	120.71 (11)
O1—C1—O2	111.52 (10)	C11—C16—H16	119.6
O1—C1—C11	108.82 (9)	C15—C16—H16	119.6
O2—C1—C11	108.88 (9)	O1—C2—C3	109.75 (12)
O1—C1—H1	109.2	O1—C2—H2A	109.7
O2—C1—H1	109.2	C3—C2—H2A	109.7
C11—C1—H1	109.2	O1—C2—H2B	109.7
C16—C11—C12	119.17 (11)	C3—C2—H2B	109.7
C16—C11—C1	120.46 (11)	H2A—C2—H2B	108.2
C12—C11—C1	120.36 (11)	C4—C3—C2	108.41 (12)
C13—C12—C11	120.79 (11)	C4—C3—H3A	110.0
C13—C12—H12	119.6	C2—C3—H3A	110.0
C11—C12—H12	119.6	C4—C3—H3B	110.0
C14—C13—C12	118.88 (11)	C2—C3—H3B	110.0
C14—C13—H13	120.6	H3A—C3—H3B	108.4
C12—C13—H13	120.6	O2—C4—C3	109.75 (11)
C15—C14—C13	121.42 (12)	O2—C4—H4A	109.7
C15—C14—Cl1	119.38 (10)	C3—C4—H4A	109.7
C13—C14—Cl1	119.20 (10)	O2—C4—H4B	109.7
C14—C15—C16	119.00 (11)	C3—C4—H4B	109.7
C14—C15—H15	120.5	H4A—C4—H4B	108.2
			
C2—O1—C1—O2	62.93 (14)	C12—C13—C14—C15	0.4 (2)
C2—O1—C1—C11	−176.94 (11)	C12—C13—C14—Cl1	−179.44 (10)
C4—O2—C1—O1	−62.80 (13)	C13—C14—C15—C16	−0.2 (2)
C4—O2—C1—C11	177.11 (10)	Cl1—C14—C15—C16	179.59 (10)
O1—C1—C11—C16	25.22 (15)	C12—C11—C16—C15	1.37 (19)
O2—C1—C11—C16	146.96 (11)	C1—C11—C16—C15	−179.55 (11)
O1—C1—C11—C12	−155.71 (11)	C14—C15—C16—C11	−0.64 (19)
O2—C1—C11—C12	−33.96 (15)	C1—O1—C2—C3	−58.71 (16)
C16—C11—C12—C13	−1.22 (19)	O1—C2—C3—C4	54.64 (17)
C1—C11—C12—C13	179.70 (11)	C1—O2—C4—C3	58.44 (15)
C11—C12—C13—C14	0.35 (19)	C2—C3—C4—O2	−54.49 (17)

### 2-(4-Chlorophenyl)-1,3-dioxane Hydrogen-bond geometry (Å, º)

*C*_g_(1) is the centroid of carbon atoms C11–C16.

**Table:**

*D*—H···*A*	*D*—H	H···*A*	*D*···*A*	*D*—H···*A*
C13—H13···O2^i^	0.95	2.66	3.4686 (16)	143
C1—H1···*C*_g_(1)^ii^	1.00	2.58	3.5282 (15)	158
C14—Cl1···*C*_g_(1)^iii^	1.74 (1)	3.49 (1)	3.9197 (15)	91 (1)

Symmetry codes: (i) −*x*+1, −*y*+1, −*z*; (ii) *x*−1, *y*, *z*; (iii) *x*+1, *y*, *z*.
